# Double Mutant Cycles as a Tool to Address Folding, Binding, and Allostery

**DOI:** 10.3390/ijms22020828

**Published:** 2021-01-15

**Authors:** Livia Pagano, Angelo Toto, Francesca Malagrinò, Lorenzo Visconti, Per Jemth, Stefano Gianni

**Affiliations:** 1 Istituto Pasteur—Fondazione Cenci Bolognetti, Dipartimento di Scienze Biochimiche ‘A. Rossi Fanelli’ and Istituto di Biologia e Patologia Molecolari del CNR, Sapienza Università di Roma, 00185 Rome, Italy; livia.pagano@uniroma1.it (L.P.); angelo.toto@uniroma1.it (A.T.); francesca.malagrino@uniroma1.it (F.M.); lorenzo.visconti@uniroma1.it (L.V.); 2 Department of Medical Biochemistry and Microbiology, Uppsala University, SE-75123 Uppsala, Sweden

**Keywords:** coupling energy, site-directed mutagenesis, interaction networks

## Abstract

Quantitative measurement of intramolecular and intermolecular interactions in protein structure is an elusive task, not easy to address experimentally. The phenomenon denoted ‘energetic coupling’ describes short- and long-range interactions between two residues in a protein system. A powerful method to identify and quantitatively characterize long-range interactions and allosteric networks in proteins or protein–ligand complexes is called double-mutant cycles analysis. In this review we describe the thermodynamic principles and basic equations that underlie the double mutant cycle methodology, its fields of application and latest employments, and caveats and pitfalls that the experimentalists must consider. In particular, we show how double mutant cycles can be a powerful tool to investigate allosteric mechanisms in protein binding reactions as well as elusive states in protein folding pathways.

## 1. Introduction

A rigorous description of any physical system demands the detailed characterization of its structural elements, as well as the quantitative investigation of its energetic components. In the case of proteins, whilst an arsenal of different experimental techniques has been developed to address the structural features of these molecules [1,2,3], the balance of forces regulating protein architecture, folding and function is much more difficult to address experimentally. In fact, such an aim is complicated by the very small changes in energy between all the different populated conformational states and their rapid rates of inter-conversion [4]. Furthermore, because protein sequences include a combination of hydrophobic (non-polar) and hydrophilic (polar) regions, the transient interactions of these regions with water molecules result in a very complex scenario and a quantitative prediction of all contributing Gibbs free energies is still elusive. 

How can we approach this problem? The so-called ‘double mutant cycles’ approach is a powerful method to measure the strength of molecular contacts between interacting side-chains [5,6,7,8,9]. This technique is generally based on the synergy between site-directed mutagenesis and quantitative measurement of biophysical properties of a protein system. Here we provide a review focused on this experimental methodology. In particular, we recapitulate the key principles of double mutant cycle analysis, exemplifying some possible applications and we discuss the method in light of published work. Furthermore, we emphasize the key caveats and pitfalls associated with double mutant cycle. 

## 2. Principles of Double Mutant Cycles and Basic Equations

In his seminal analysis of the theory of heredity [10], William Bateson observed that, for several genetic features, the concept of ‘dominant’ and ‘recessive’, previously introduced by Mendel, was inadequate. In fact, in some cases, it was clear that the effect of a mutation in given gene was severely influenced by one or more other genes. In these cases, the term ‘epistasis’ was suggested, reflecting the condition whereby the phenotypic manifestation of a mutation is dependent on the genetic background in which it appears [11,12]. Analogously, ever since the early days of protein engineering, it was clear that some features of amino-acid side chains in regulating protein function were dependent on other side chains. In fact, only two years after the purification of the very first site-directed mutant, performed on the tyrosyl-tRNA synthetase in 1982 [13], it was observed on the same enzyme that the role of two residues in the active site was affected by the conformational change introduced by a threonine to proline mutation in a distal position, thereby introducing the concept of ‘coupling’ [14]. 

It is of critical importance to define quantitatively the extent of coupling between two interacting amino acids as well as to introduce a feasible methodology to measure it, the double mutant cycle [5,6,7,8,9]. In principle, a double mutant cycle assumes that when a perturbation is introduced in two non-interacting residues, X and Y, the effect of each single perturbation is additive in a double mutant where both perturbations are present. Thus, the change in free energy upon mutation of X, associated with any structural or functional behavior of a given protein, may be expressed as ΔΔG_P-XY→P-Y_. Analogously, the change in free energy associated with the residue Y will be equal to ΔΔG_P-XY→P-X_. If the mutations are independent of each other, then a double mutant of X and Y will display as:

ΔΔG_P-XY→P_ = ΔΔG_P-XY→P-Y_ + ΔΔG_P-XY→P-X_ (1)

**Figure 1:**
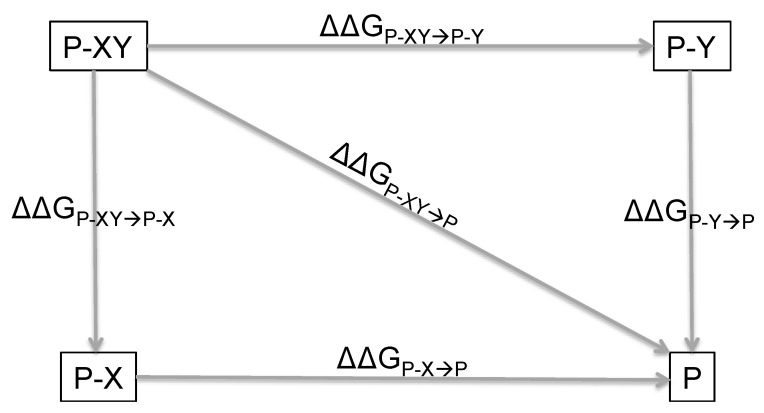
Schematic representation of a double mutant cycle. P-XY is the wild-type protein with the two residues X and Y. P-X and P-Y are the variants where Y and X are mutated, respectively, and P is the corresponding double mutant. The change in free energy upon mutation of X (or Y) is equal to ΔΔG_P-XY→P-Y_ (or ΔΔG_P-XY→P-X_) and ΔΔG_P-XY→P_ is the change in free energy of the double mutant.

Conversely, a non-zero value of:

ΔΔΔG_XY_ = ΔΔG_P-XY→P_ − ΔΔG_P-XY→P-Y_ − ΔΔG_P-XY→P-X_ (2)

would correspond to the free energy of interaction between the X and Y and would therefore correspond to the coupling free energy of X and Y with respect to the probed structural or functional property of the protein. The ΔΔΔG_XY_ measures therefore the energetic strength of the interaction between positions X and Y and, when its value is different from zero, it represents the interaction (or coupling) energy between the two residues. A scheme summarizing the basic principles and equations of double mutant cycles is reported in Figure 1.

## 3. The Double Mutant Cycle: Strengths, Caveats, and Pitfalls

When introducing an experimental technique, it is of particular importance to highlight its major advantages, as well as the caveats and pitfalls. Thus, what is the advantage to perform double mutant cycles as compared to single site mutagenesis? When mutating an amino acid side-chain into another, the change in free energy upon mutation ΔG_N-N′_ is in fact the sum of different components [15]: (i) The change in free energy of covalent bonds, ΔG_cov_; (ii) that arising from the changes in noncovalent bonds located at the site of mutation, ΔG_noncov_; (iii) any additional free energy changes related to a reorganization of the protein, ΔG_reorg_; and (iv) changes in solvation energy, ΔG_solv_. When performing folding and/or binding experiments, the ΔG_cov_ is conserved in all states and, therefore, it cancels out in the analysis. On the other hand, ΔG_noncov_ is generally assumed to be the dominant contributor to ΔG_N-N′_. However, the contribution of the four ΔG values may differ for different states. For example, the values of ΔG_reorg_ and ΔG_solv_ may be different in native and denatured states for a folding reaction, such that ΔG_N-N’_-ΔG_D-D′_ will report not only on the energetics at the mutation site ΔG_noncov_. This effect will complicate the analysis of the experimental data. A similar situation may occur for bound and free states for a bimolecular interaction.

To exemplify this scenario, let us consider the binding between a protein and a ligand:

P_N_ + L = P_N_L (3)

When and if a site-directed mutation is introduced in P_N_, the folding stability of the protein could be affected, such that its native state is destabilized. Thus, by considering the scheme:

P_D_ = P_N_ + L = P_N_L (4)

In the latter case, binding is considered in conjunction with a large conformational change, such as the (un)folding of the protein. In this case, it is clear that the apparent *K_D_* would be affected by the presence of a linked equilibrium, such that:

(5) $$K_{D}^{app}=K_{D}\frac{1}{1+\frac{1}{K_{D-N}}}$$

Thus, if the mutation affects the stability of the native state such that a significant population of the protein is shifted towards the denatured state, the apparent binding constant is also affected; even in cases in which the mutation would not perturb the binding interface at all. In those cases, ΔG_reorg_ and ΔG_solv_ are dominant over the value of ΔG_noncov_, and any analysis of the local effect of the mutation is prevented.

By considering these premises, it is clear that the double mutant cycle, which relies on calculating the difference between the effects on the double mutant over the two single variants, is extremely powerful. In fact, even in cases in which the mutation results in structural shifts of the protein, as exemplified above, those found in the single mutants are usually present in the double mutant and, therefore, their energetic effects in the double mutant cycle are cancelled out [16,17,18]. These considerations reinforce the importance of this method as a tool to investigate directly the energetics of interaction between two residues.

Due to the complexity of site directed mutagenesis, it is important to stress that conservative mutations should be used to probe binding or folding [19]. Values of ΔG_reorg_ and ΔG_solv_ for conservative mutations are more likely to be lower than the value of ΔG_noncov_, which, as briefly discussed above, tends to simplify the analysis. Additionally, since the coupling free energy arises from the difference between the value obtained for the double mutant minus the sum of the values obtained from the single mutants, it might be difficult to calculate a reliable value of ΔΔΔG_XY_ when the effect of one mutation is very large such that limitations in the biophysical method result in a large experimental error. For example, isothermal titration calorimetry usually works best in the range 100 nM–1 μM and mutations resulting in a 100-fold destabilization will lead to a large experimental error. 

Over and above the theoretical basis of this type of analysis, there are also some practical issues that demand additional consideration. In fact, it is a good advice to choose the mutations properly. Since the double mutant cycle technique is aimed at measuring the free energy of interactions between two residues, it is recommended to keep the two terms ΔG_reorg_ and ΔG_solv_ as low as possible. Consequently, since mutations of large hydrophobic side chains may lead to very large ΔG_reorg_ and ΔG_solv_, these are not well suited for double mutant cycles. Thus, in analogy to the Φ value analysis in protein folding studies [19], it is recommended to mutate hydrophobic side chains, introducing small side chain deletions and without altering the stereochemistry (i.e., Ile→Val→Ala→Gly; Leu→Ala→Gly; Thr→Ser; Phe→Ala→Gly). Additionally, analogously to what is normally suggested in the case of the Φ value analysis in protein folding, a reliable dataset is generally based on a large number of site-directed mutants, allowing to probe pairwise residue interactions encompassing a relevant fraction of the protein structure. 

After the mutations are introduced, it is critical to measure the changes in free energy accurately. This task may be achieved by employing the standard spectroscopic methodologies that are classically used in affinity and/or stability measurements for proteins. These techniques may span from equilibrium titration with spectroscopic techniques such as absorbance, fluorescence, circular dichroism, etc., to other methods, such as calorimetry.

## 4. Double Mutant Cycles to Understand Intramolecular Interactions

The invention of double mutant cycles was associated with the quantification of intramolecular interactions in proteins. In their pioneering work on protein engineering Winter, Fersht, and co-workers observed that, in the case of a tyrosyl-tRNA synthetase, a variant in which a threonine residue was mutated into proline increased the affinity of the enzyme for ATP [13]. By making mutants of the proline-containing enzyme at two other positions directly involved in ATP binding, it was possible to show that the presence of the proline improves the strength of one of these contacts. On the basis of these observations, it was proposed that the propagation of a structural change in an enzyme induced by mutation could be explored by the use of further mutations, thereby introducing the double mutant cycle methodology as a unique tool for the investigation of intramolecular interactions in proteins.

Following this original work, several protein systems of known structure have been subjected to double mutant cycles [6]. In these cases, the method represents a powerful tool to unveil the details of distinct interactions in protein stability and function. In this context, it is worth to highlight the employment of double mutant cycles to clarify the role of surface and buried salt bridges in protein stability, as well as charge-aromatic interactions. Importantly, the cycles have also been used in more complex approaches with three interacting residues, implying the design of multidimensional cycles. Barnase [20,21,22], staphylococcal nuclease [23,24], and lambda repressor [25,26] are three examples of proteins subjected to double mutant cycle analysis.

**Figure 2:**
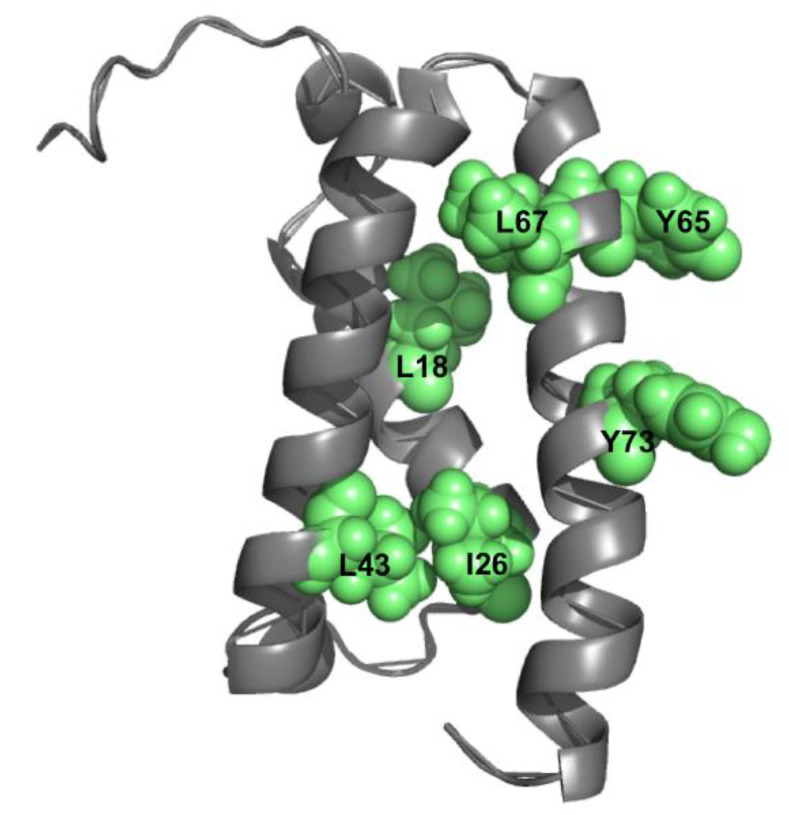
Structural distribution of residues L18, I26, L43, Y65, L67, and Y73 (green spheres) in the KIX domain (PDB 2AGH). The analysis of folding *m* values, in combination with double mutant cycle analysis, allowed to characterize, from a thermodynamic perspective (i.e., measuring ΔΔΔG values), the role of these residues in stabilizing non-native interactions in the denatured state of KIX. With the exception of I26 and L43 that are in direct contact, residues displaying significant coupling free energies are located in different regions of the domain (see [28] for details).

Over and above the cases in which double mutant cycles have been employed to address intramolecular interactions in known structures, it is important to note that, in some context, the approach can be used to investigate elusive states that are difficult to characterize experimentally. The KIX domain is a globular domain that is a part of a large coactivator protein called CBP [27]. The three-dimensional structure of KIX consists of three α-helices and two short 3_10−_ helices (Figure 2). A detailed characterization of its folding pathway revealed that, whilst the protein appears to fold in a simple two-state manner, its denatured state retains a considerable amount of structure [28], as probed by the analysis of the so-called *m* values, a set of parameters related to the change in accessible surface area upon (un)folding [29]. To address the structural details of the residual structure in the denatured state of KIX, double mutant cycles were performed [28]. In particular, by focusing primarily on the single variants that had a major effect on the folding *m* values, a series of double mutants were produced and characterized. Interestingly, the folding characterization of these mutants allowed mapping the presence of distinct non-native interactions in the denatured state, as well as in measuring their strength in terms of ΔΔΔG. A similar analysis of structure in the denatured state was also performed on the C-terminal domain of nucleophosmin by conducting folding and unfolding experiments on several site-directed variants at different experimental conditions [30].

## 5. Protein Binding and Allostery

Ever since the pioneering work of Kendrew and Perutz, it became clear that a deep understanding of proteins’ structure and function would depend on the rigorous description of their dynamic properties. In fact, only the identification of the R and T quaternary states of haemoglobin provided a structural framework to understand its cooperative and allosteric behavior, pinpointing the role of residues ‘other’ (from the Greek *allo*-) than those in the binding site in regulating function [31]. In this context, when studying the binding of two molecules, it is clear that double mutant cycles may provide a straightforward method to analyze binding and allostery [16,32]. When studying a number of i mutants in the A protein, binding to j mutants of the B protein, it is possible to address i × j double mutant cycles and define their interactions quantitatively. In practical terms, in those cases, it would be required to perform the experiments: (i) on the mutant of protein A versus the wild-type form of the protein B; (ii) on the wild-type of protein A versus the mutant form of the protein B; (iii) on all combinations of the mutant variants of both proteins. If the changes in free energies are not additive, the probed residues are energetically coupled. 

The interaction between barnase and barstar represents a paradigm example of the use of double mutant cycles to understand protein-protein binding. In this case, several site-directed mutants were analyzed providing a detailed description of the energetics of such interactions [18,33]. In particular, it was found that the coupling free energy generally depends on the distance between the probed residues. Whilst residues closer than 7 Å tend to interact co-operatively, at greater distances the changes in free energy upon mutations become additive. However, it was found that the interaction network between functional residues is not always trivial [34,35,36,37], such that they can be directly deduced from the crystal structure. For example, the salt-bridge between Lys27 in barnase and Asp39 of barstar was found to be very strong, while that between Glu76 of barstar and Arg59 of barnase is relatively weak, showing the importance of the local environment for binding [38], as well as the need to complement the structural data with biophysical experiments.

The double mutant cycle approach has been subsequently employed on other protein systems, with the specific aim to quantify the interaction networks between interacting protein systems. Relevant examples include coiled-coil XGCN4-p1 [39], as well as characterization of the allosteric network of different PDZ [40,41,42,43] and SH3 domains [44]. In all these cases, it was possible to identify energetic coupling between residues that were not necessarily in direct contact in the three-dimensional structure of the respective protein complexes, confirming the concept that allosteric networks tend to follow intricate pathways that demand a careful experimental investigation.

**Figure 3:**
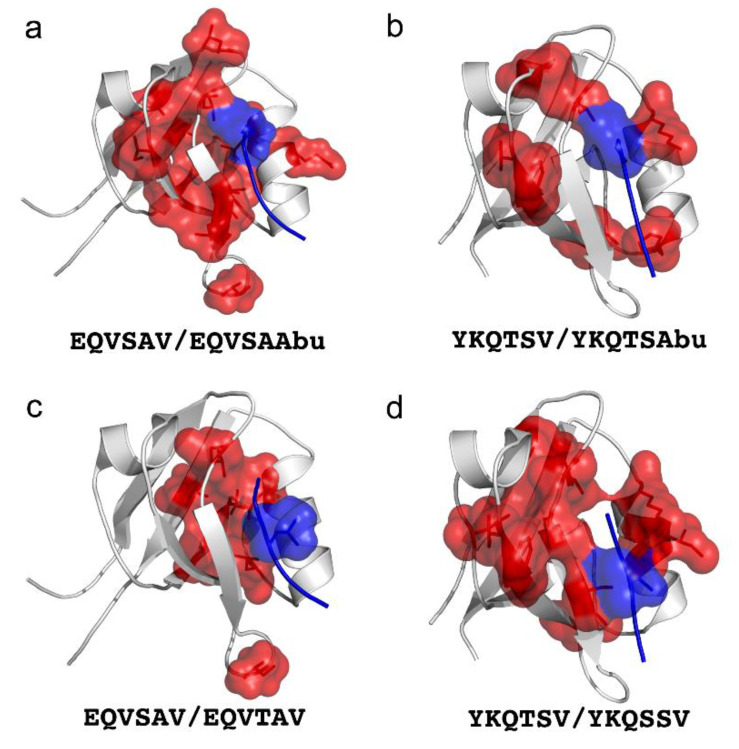
Graphic representation of the different allosteric networks of PDZ domain-ligand complexes of protein tyrosine phosphatase Basophil-like PDZ2 (**a** and **c** panels) and PSD-95 PDZ3 (**b** and **d** panels) with their natural ligand (showed below each panel). All the mutated residues are represented as spheres both in the ligand (blue) and in the PDZ domain (red). The peptide ligand mutations Val→Abu (deletion of a γ-methyl group from Val), Ser→Thr or Thr→Ser were chosen because they interact with the binding pocket of PDZ domains.

Out of the examples highlighted above, it is particularly interesting to discuss some of the conclusions that have been drawn on PDZ domains [40,41,42,43]. In fact, the experimental data suggested that, in the majority of cases, a positive coupling energy for the Val_0_ to Abu substitution was observed in both PDZ2 from PTP-BL and PDZ3 from PSD95 (Figure 3), although the absolute values were variable. Thus, the effect of the mutations in the protein on binding was more pronounced when the wild-type peptide was used, as compared to that in which a methyl group was removed from position 0. This intriguing finding suggested that the entire PDZ structure could been under selective pressure to optimize the binding of its physiological ligand. Sequence variation in the protein domain is therefore not neutral to peptide binding, strongly indicating that selectivity by the domain is not solely determined by the subset of residues directly involved in ligand binding. Importantly, this finding appears to support a scenario whereby the cross-talk between binding sites and remote residues may be used to fine tune target selectivity, and possibly to decrease the cross-reactivity between homologous PDZ domains.

The complex nature of the functions of proteins often demands their structure to be composed of multiple domains. In these cases, it is very frequent that function and binding may depend on the interdomain architecture and dynamics, such that the individual domains in isolation display different features compared to when they are present in multidomain constructs. While this concept is widely accepted, our detailed knowledge about the effects of supertertiary structure in folding and function is still limited. In this context, double mutant cycles proved very useful. An extensive double mutant cycle analysis of a PDZ domain, both in isolation and in the context of a supramodule comprising the PDZ domain, an SH3 and a GK domain, showed that allosteric networks are highly sensitive to the supertertiary structure [45]. In particular, it was found that the presence of the SH3–GK tandem resulted in strong coupling from the bound peptide ligand to the β1β2 loop, β2β3 loop, and α3 helix that was not observed with the single PDZ3 domain. These findings prompted the authors to reinforce the importance of extending double mutant cycle experiments to multidomain systems. 

## 6. Double Mutant Cycles In Silico

The double mutant cycle methodology generally relies on the design, production and characterization of protein mutant variants. Nevertheless, an interesting application of this method is an in silico analysis only. Horovitz and co-workers, who investigated the interaction between two cysteine residues, namely Cys137 and Cys518, in the *Escherichia coli* chaperonin GroEL, first introduced this approach [46]. By performing a multiple sequence alignment on proteins belonging to the Hsp60 family, they found that naturally-occurring variations at positions 137 and 518 were likely to be coordinated. An experimental validation of this interaction further substantiated this analysis and suggested that the study of the co-variance of residues at different positions may represent a valuable method to detect interactions in protein systems.

In a seminal study published in Science, Lockless and Ranganathan extended the approach to a larger scale [47]. By using the PDZ domain family as a model system, the authors investigated systematically the evolutionary coupling between the different positions of the domain. The principle of the method is that a statistical coupling between two sites, i and j, may be defined as the degree to which amino acid frequencies at site i change in response to a perturbation of frequencies at another site, j. Then, by focusing on residue His76, which is in an important position in defining the specificity of PDZ domains, a set of energetically coupled positions for the binding site was identified, including unexpected long-range interactions. Whilst extremely interesting, however, this study was subsequently reassessed and it was found that the energetic coupling was not a special feature of the coevolved network of residues in PDZ domains [48,49]. In addition, as mentioned, in the context of the PDZ3-SH3-GK supramodule the experimentally determined allosteric network for PDZ3 is different from that in isolation [50]. Thus, statistical coupling from sequence analysis is not necessarily a reporter of energetic coupling and allostery and must, therefore, be supported with extensive experimental data.

## 7. Double Mutant Cycles by Native Mass Spectrometry

A very interesting application of double mutant cycles has been recently implemented by studying the formation of protein-protein interactions via native mass spectrometry [51]. The intrinsic power of this technique lies in the ability to transfer protein complexes to the gas phase, without altering their native state [52,53,54]. Hence, by measuring a single high-resolution native mass spectrum and determining the intensities of the complexes formed by the two wild-type proteins, the complex of each wild-type protein with a mutant protein, and the complex of the two mutant proteins, it is possible to obtain the pairwise interaction energies between the two mutated residues with great precision. In fact, it can be demonstrated that native mass spectrometry both circumvents the determination of individual binding constants, which are of course prone to experimental error, and is independent of the concentration of the free unbound species [55]. Remarkably, the method may be employed directly on crude cell lysates [56], which further simplifies the experiments and interpretations. In fact, in these cases, the purification of the mutant variants may not be required, which simplifies the experimental approach consistently. 

Double mutant cycles by mass spectrometry have been successfully employed to study the binding between the proteins E9 and Im2 [51] and, more recently, to understand the dimerization of SOD1 in naturally-occurring mutants associated with ALS. The authors analyzed the coupling constants by co-expressing wild-type SOD1 with ALS-causing mutations [57]. The result is a quantitative measurement of the inherent preference of these variants to form homo-dimers (mutant-mutant or wild type-wild type) rather than heterodimers (mutant-wild type). Of exceptional interest, the analysis successfully demonstrated that heterodimerization preference of SOD1 in ALS-causing mutations correlates with the reported average duration of the disease. Their findings suggest that heterodimerization of mutant variants of SOD1 is directly involved in the development of ALS and provide another clear example of how the initial events of self-assembly represent a critical point in the intimate link between protein misfolding and disease [58]. 

## 8. Conclusions

The double mutant cycles methodology represents a very powerful technique offering the tantalizing possibility to address directly the strength of pairwise interactions in proteins. As exemplified in the case of PDZ domains, the analysis of the sign of the observed energetic coupling may be used to describe protein selectivity in conserved homologous protein-protein interaction domains. Furthermore, in conjunction with other techniques, it may represent a future tool to address ambitious tasks, such as the understanding of structure of metastable states as well as the description of aggregation phenomena. With the specific aim to help the experimentalist, we have recapitulated here the key features, along with the associated caveats and pitfalls, as well as some key examples of applications.
